# A Retrospective Study on the Incidence, Mortality, and Risk Factors of Hodgkin Lymphoma in Pakistan

**DOI:** 10.7759/cureus.84657

**Published:** 2025-05-23

**Authors:** Seema Shafiq, Shahzadi Noor Fatima, Anita Zahid, Tania Khattak, Seherish Khan Abbasi, Muhammad Rehan Mumtaz

**Affiliations:** 1 Oral Pathology, Islamic International Dental College and Hospital, Riphah International University, Islamabad, PAK; 2 Pathology, Liaquat National Hospital, Karachi, PAK; 3 Oral Medicine, Hamdard University Dental Hospital, Karachi, PAK; 4 Histopathology, Nowshera Medical College/Qazi Hussain Ahmad Medical Complex, Nowshera, PAK; 5 Oral and Maxillofacial Surgery, Baqai Medical University, Karachi, PAK; 6 Internal Medicine, Aziz Bhatti Shaheed Teaching Hospital, Gujrat, PAK

**Keywords:** hodgkin lymphoma (hl), mortality index, patients, risk factors, satisfaction

## Abstract

Background: Hodgkin lymphoma (HL) is a type of cancer that affects lymphatic tissues and is marked by the presence of Reed-Sternberg cells, which are large, abnormal lymphocytes. Involvement of the head and neck region is a prominent feature in HL, often serving as the initial clinical presentation of the disease.

Objective: The main objective of the study is to find the incidence, mortality rate, and risk factors for HL in the Pakistani population.

Methods: A retrospective study was conducted with 50 patients diagnosed with HL across three cancer treatment centers in Pakistan. Data were collected from medical records and structured patient interviews, examining demographic factors, clinical stage, treatment types, and risk factors, including Epstein-Barr virus (EBV) infection. Statistical analyses assessed correlations between risk factors, disease stage, and mortality.

Results: The highest incidence was observed in young adults (18-30 years), with males representing 65% of cases. Mortality was significantly higher among patients diagnosed at advanced stages (stage III and IV), particularly in rural areas with limited access to treatment. EBV infection and low socioeconomic status were associated with late-stage diagnosis and poorer survival rates. Patients receiving combined chemotherapy and radiotherapy showed better one-year survival outcomes.

Conclusion: HL in Pakistan is characterized by late-stage diagnosis, high mortality, and socioeconomic disparities affecting access to care. Addressing these challenges through improved awareness, early screening, and accessible treatment could reduce mortality and improve outcomes.

## Introduction

Hodgkin lymphoma (HL) is a cancer of the lymphatic system that mainly consists of Reed-Sternberg cells. The heterogeneity in the histologic findings of HL arises from the variable nature of the malignant cells and the response of the host [1]. The prevalence of HL among total cancer cases in Pakistan creates difficulties in its management due to healthcare demographic conditions and system dimensions [2]. In the first stage, HL usually affects the head and neck regions in patients. Since the head and neck are mostly affected by HL, cervical lymph node swelling occurs in 60% to 80% of patients and is generally their first symptom [3]. In early Hodgkin's disease, the anterior and posterior neck, plus the supraclavicular and submandibular lymph nodes, often become enlarged and do not hurt [4]. In advanced cases of HL, patients may experience direct extension of lymphoma into tonsillar tissue and nasopharyngeal structures or glands and salivary glands. When bulky disease compresses nearby structures, it creates symptoms that consist of dysphagia and hoarseness along with facial swelling. Early diagnosis of HL depends heavily on head and neck manifestations because health professionals who specialize in otolaryngology need to identify suspicious lymphadenopathy so biopsy examinations can lead to prompt diagnosis and better clinical outcomes [5]. The survival rate of HL in Pakistan relies on treatment accessibility and healthcare services. Worldwide statistics position HL as a carcinoma, which shows high curability rates because developed nations typically reach a five-year survival rate of 85%. The continuous high mortality rates in Pakistan result from late medical diagnosis combined with inadequate tertiary healthcare institutions and socioeconomic challenges [6]. The diagnostic process in Pakistan reveals advanced cases of HL since the determined stages make treatment less effective, with lower survival probabilities. Geographical distribution works as a mortality factor because Karachi and Lahore provide advanced cancer treatment services, which outperform facilities in remote regions [7]. The risk elements for HL in Pakistan stem from genetic and environmental circumstances and infected conditions. The development of HL worldwide depends heavily on infection with the Epstein-Barr virus (EBV). Scientific research indicates a strong positive link exists between EBV and HL development and especially affecting the mixed cellularity type. EBV occurs frequently in Pakistan, so it plays an essential role in HL development according to research [8]. Among young individuals, the immune system responses, which connect to EBV, may help explain the development of the disease. Genetic susceptibility to HL has been identified in other studies, but insufficient evidence exists regarding the Pakistani population [9]. Predicting and characterizing HL in Pakistan requires consideration of factors based on social demographics, which include socioeconomic status (SES) elements. Patients display poor recognition of HL indications since painless lymph node swelling joins fever and weight loss symptoms and night sweats, leading to difficulties when handling a diagnosis [10].

Objective

The main objective of the study is to find the incidence, mortality rate, and risk factors for HL in the Pakistani population.

## Materials and methods

This retrospective study was conducted at Islamic International Dental College & Hospital, Riphah International University, from June 2023 to June 2024. The study involved 50 patients diagnosed with HL, selected through purposive sampling. Inclusion criteria required participants to be diagnosed with HL, regardless of subtype or disease stage, within the past year and to be receiving or have received treatment at one of the study centers. Exclusion criteria included patients under 15 years of age and those with incomplete medical records or other concurrent malignancies.

Data collection

Data collection involved both retrospective review of patient medical records and structured patient interviews. Medical records were reviewed to gather information on demographic details such as age, gender, and socioeconomic status; disease-related parameters, including stage and histological subtype at diagnosis; treatment modalities and their duration; and clinical outcomes, including survival status when available. Structured questionnaires were used to obtain additional data on lifestyle habits, family history of malignancies, possible environmental exposures, and previous infections relevant to HL risk factors, particularly EBV. Informed consent was obtained from all participants for both access to medical records and participation in interviews, in line with ethical standards and institutional review board approval.

Data analysis

Data were analyzed using SPSS version 21 (IBM Corp., Armonk, NY). Incidence was calculated based on the total number of diagnosed HL cases in the sample. Mortality rate was assessed as the proportion of patients who died within one year of their initial diagnosis. Further analyses stratified incidence and mortality by patient age, gender, and clinical stage at presentation to identify potential trends and associations. A p-value of less than 0.05 was considered statistically significant.

## Results

Data were collected from 50 patients, with a mean age of 31.45 ± 5.68 years, ranging from 18 to 60 years. Males represented 33 (66%) of the patients, while females accounted for 17 (34%), indicating a higher prevalence among males. The majority of patients (30, 60%) were from rural areas, highlighting potential challenges related to healthcare access compared to the 20 (40%) from urban settings. EBV positivity was observed in 28 (56%) patients, while 32 (64%) belonged to a low socioeconomic background. Environmental exposure was reported in 15 (30%) patients, and a family history of lymphoma was present in six (12%) (Table 1).

**Table 1:** Demographic and baseline values. Continuous variables are presented as mean ± SD. Categorical variables are presented as N or %, as appropriate. A p-value of <0.05 is considered statistically significant unless otherwise specified.

Variable/risk factor	Value (n, %)
Total patients	50 (100%)
Mean age (years)	31.45 ± 5.68
Age range (years)	18–60
Gender	
– Male	33 (66.0%)
– Female	17 (34.0%)
Residence	
– Urban	20 (40.0%)
– Rural	30 (60.0%)
Epstein-Barr virus (EBV) positive	28 (56.0%)
Low socioeconomic status (SES)	32 (64.0%)
Family history of lymphoma	6 (12.0%)
Environmental exposure (e.g., smoke/industry)	15 (30.0%)
History of immunosuppression	4 (8.0%)

The stage at diagnosis among the patients revealed that 10 (20%) were diagnosed at stage I and 15 (30%) at stage II, indicating that half of the patients were identified in early stages. However, 15 (30%) patients were diagnosed at stage III and 10 (20%) at stage IV, suggesting a substantial proportion of cases were identified in advanced stages (Table 2).

**Table 2:** Disease stage at diagnosis. A p-value of <0.05 is considered statistically significant.

Stage at diagnosis	Number of patients (n)	Percentage (%)	95% confidence interval	Margin of error (%)
Stage I	10	20.0%	9.8% – 30.2%	±10.2%
Stage II	15	30.0%	17.1% – 42.9%	±12.9%
Stage III	15	30.0%	17.1% – 42.9%	±12.9%
Stage IV	10	20.0%	9.8% – 30.2%	±10.2%

Among the reported symptoms, 40 (80%) of patients experienced painless swelling of lymph nodes, making it the most common presenting symptom of HL. Additionally, 25 (50%) reported fever, 30 (60%) experienced night sweats, and 20 (40%) noted weight loss (Table 3).

**Table 3:** Symptom presentation at diagnosis.

Symptom type	Number of patients reporting	Percentage (%)
Painless swelling of lymph nodes	40	80
Fever	25	50
Night sweats	30	60
Weight loss	20	40

The overall mortality rate for HL among the study population was 24%, with 12 deaths recorded. Mortality was notably lower in early stages (n = 2, 8% for stages I & II) compared to advanced stages (n = 10, 40% for stages III & IV), reflecting the impact of disease progression on outcomes. Additionally, rural patients experienced a higher mortality rate of nine (30%) compared to three (15%) in urban patients, highlighting disparities in access to healthcare and emphasizing the need for targeted support and resources in rural areas to improve survival rates (Table 4).

**Table 4:** Mortality rate by disease stage and location.

Category	Number of patients (n)	Number of deaths (n)	Mortality rate (%)
Total study population	50	12	24.0%
Early stages (I & II)	25	2	8.0%
Advanced stages (III & IV)	25	10	40.0%
Rural patients	30	9	30.0%
Urban patients	20	3	15.0%

## Discussion

This study highlights important findings regarding the mortality and risk factors of HL in Pakistan. These findings reflect the difficulties that local and international healthcare providers face in managing this cancer. The information gleaned from 50 patients sheds light on how the disease's progression and outcomes are influenced by a variety of factors, including demographics, accessibility to healthcare, socioeconomic status, and specific risk factors like EBV infection. Pakistan's healthcare infrastructure and public health strategies will be significantly impacted by these findings. The study observed that HL in Pakistan has a higher incidence in younger populations, particularly in the 18-30 years age group [11]. This is in line with global trends because, in contrast to other cancers, HL has a higher incidence in young adults and is characterized by distinct biological and environmental factors. Genetic predispositions, lifestyle factors, and the prevalence of infections in the region could contribute to the increased incidence in this age group [12]. Age-specific awareness programs, early screening, and a focus on reducing risk exposure among younger people are all necessary, as this trend demonstrates. This study's mortality rates highlight the difficulties in diagnosing HL in its early stages in Pakistan [13]. When compared to patients who were diagnosed at earlier stages, those diagnosed at advanced stages (stage III and IV) had a mortality rate that was significantly higher. This could be because early healthcare-seeking behavior is hindered by socioeconomic barriers, delays in diagnosis, and limited access to specialized cancer treatment [14]. The high mortality rates among rural patients further underscore these access issues, emphasizing the need for improved healthcare infrastructure in rural and underserved areas. In this study, EBV was found to be a significant risk factor, as a significant number of EBV-positive patients presented with advanced HL [15]. Poorer outcomes were seen in patients from lower-income backgrounds, who frequently encounter obstacles to timely diagnosis and treatment [16]. The lack of insurance, financial constraints, and awareness of HL symptoms were all factors. This suggests that community-based health education initiatives, financial support for cancer treatment, and more accessible healthcare options are required. Patients from all walks of life would be able to benefit from such interventions by having access to timely and affordable cancer care [17]. According to the findings, the type of treatment has a significant impact on survival outcomes. One-year survival rates were better for patients who received radiotherapy in addition to chemotherapy than for those who delayed treatment or received chemotherapy alone [18]. Poorer survival rates were found to be associated with delayed treatment initiation, frequently as a result of financial and logistical difficulties [19]. Accordingly, healthcare policies that support immediate access to therapy and reduce wait times are required. Investing in affordable, timely, and comprehensive treatment options for HL could substantially improve survival rates in Pakistan. To reduce the mortality associated with HL, it is essential to implement healthcare policies that encourage prompt and ongoing treatment regardless of socioeconomic status [20].

However, this study has several limitations. First, the sample size was relatively small (N = 50), which may affect the generalizability of the findings to the wider population. Second, the study was conducted at a single center, potentially introducing selection bias and limiting the diversity of patient backgrounds. Third, data on EBV status and treatment adherence were based on retrospective reviews and may lack full clinical validation. These limitations suggest that larger, multicenter prospective studies with more robust statistical controls are needed to confirm and expand on these findings.

## Conclusions

It is concluded that HL in Pakistan primarily affects younger populations, with high mortality rates linked to late-stage diagnoses, limited healthcare access, and socioeconomic barriers. Effective interventions, such as improved awareness, early screening, and accessible treatment, are essential for reducing mortality and improving outcomes for HL patients in Pakistan. Addressing these challenges through targeted policies could substantially improve survival rates and healthcare equity.
